# A rare case of cytomegalovirus, scedosporium apiospermum and mycobacterium tuberculosis in a renal transplant recipient

**DOI:** 10.1186/1471-2334-14-259

**Published:** 2014-05-14

**Authors:** Manish Rathi, Srikant Gundlapalli, Raja Ramachandran, Sandeep Mohindra, Harsimran Kaur, Vivek Kumar, Harbir Singh Kohli, Krishan Lal Gupta, Vinay Sakhuja

**Affiliations:** 1 Department of Nephrology, Postgraduate Institute of Medical Education & Research, Chandigarh 160012, India; 2 Department of Mycology, Postgraduate Institute of Medical Education & Research, Chandigarh, India; 3 Department of Neurosurgery, Postgraduate Institute of Medical Education & Research, Chandigarh 160012, India

**Keywords:** *Cytomegalovirus*, *Scedosporium apiospermum*, Renal transplant, Brain abscess, Post transplant tuberculosis

## Abstract

**Background:**

Renal transplant recipients are at high risk of developing multiple infections, often concomitantly because of their immunocompromised status. Post renal transplant infections are often elusive and require extensive evaluation for proper diagnosis and treatment. A high index of suspicion is required and an attempt should be made to confirm the microbiological diagnosis from each site involved to rule out multiple infections.

**Case presentation:**

We report a 50-year-old female, a renal allograft recipient who presented with left hemiplegia, esophageal ulcers and fever 3 months after her transplant. Esophageal biopsy revealed *Cytomegalovirus* (CMV) inclusions and the whole blood quantitative CMV polymerase chain reaction (PCR) was positive. Neuroimaging showed a brain abscess, stereotactic biopsy from which revealed *Scedosporium apiospermum* on fungal culture. Her tacrolimus and mycophenolate were stopped and she was managed with intravenous ganciclovir and voriconazole. With these measures, she showed marked improvement in her general and neurological condition. Two months later, she developed recurrence of fever with dry cough. Radiological investigation revealed a cavitating lung lesion, a needle aspiration from which demonstrated acid-fast bacilli. She was started on antituberculous treatment. With these measures, she recovered completely and maintained good graft function despite being on only prednisolone 10 mg once a day.

**Conclusion:**

Although CMV disease is not uncommon in the first three months post transplant, *Scedosporium* is a rare cause of brain abscess. On the other hand, tuberculosis is common in transplant recipients, especially in developing countries, like India. However, this is the first case report of occurrence of these three infections in the same patient, demonstrating the importance of a good microbiological work-up from each site involved in immunosuppressed subjects.

## Background

Renal transplantation is the best option for patients with end-stage kidney disease, more so for those in developing countries where facilities for maintenance dialysis are inadequate [1]. Although survival rates after renal transplantation have improved considerably, infections remain a major cause of morbidity and mortality in countries with poor socioeconomic conditions. Studies from such countries have reported infection rates in the range of 50-80% and a mortality rate of 20-60%, particularly with severe opportunistic and fungal infections [2,3]. Various bacterial, viral, fungal and mycobacterial infections may occur simultaneously or sequentially and tissue sampling is often the only mode of conclusive diagnosis. *Cytomegalovirus* (CMV) and mycobacterial infections are common and well described post renal transplant infections, particularly in the period of intense immunosuppression; however, *Scedosporium* is a rare fungal pathogen affecting renal transplant recipients. We report a rare case of CMV, *Scedosporium apiospermum*, and *Mycobacterium tuberculosis* infection in a renal transplant recipient, all of which were managed successfully.

## Case presentation

**Figure 1:**
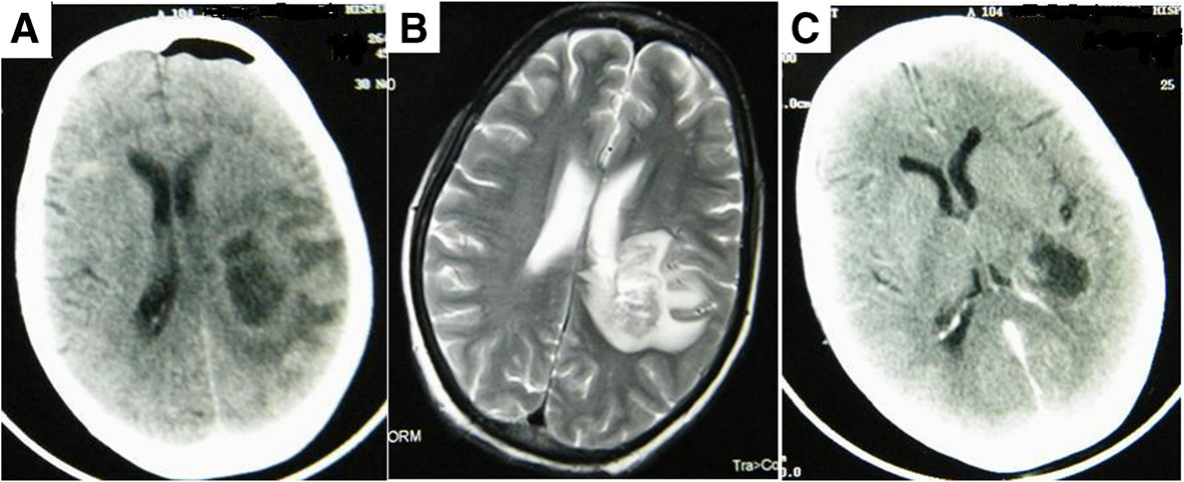
**Radiology of head. (A)** NCCT head showing a large hyperdense lesion in the right temporo-parietal region with peri-lesional edema, which is confirmed on MRI **(B)**. Significant reduction in size of the lesion after drainage and antifungal therapy on repeat NCCT head **(C)**.

**Figure 2:**
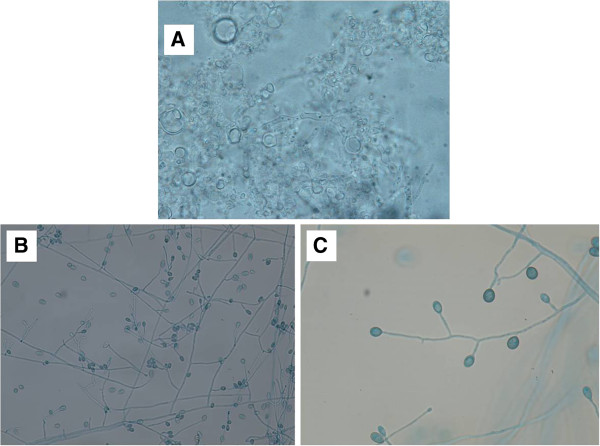
**Mycological work-up of brain abscess aspirate. (A)** KOH (potassium hydroxide) mount showing thin septate hyphae in the pus from brain abscess, **(B)** Lactophenol cotton blue mount of culture material showing numerous single-celled, broadly clavate to ovoid conidia, 4–9 x 6–10 mm, rounded above with truncate bases, borne singly or in small groups on elongate, simple or branched conidiophores or laterally on hyphae **(C)**.

A 50-year-old housewife was diagnosed to have end-stage renal disease of uncertain etiology. After being on hemodialysis for one year, she underwent living unrelated renal transplantation at another centre with basiliximab induction followed by triple drug immunosuppression with tacrolimus (trough level 6.7-8.8 ng/mL), mycophenolate mofetil (1 gm twice daily) and prednisolone (5 mg daily). The pretransplant CMV sero-status of the donor and recipient was not known and she was not on prophylaxis for CMV. Three months post transplant, she was admitted with fever, headache, oral ulcers and dysphagia of one week duration and soon after admission she developed slurring of speech with drowsiness and weakness of left upper and lower limbs. During the preceding three months, she had maintained normal graft function and did not receive any anti-rejection therapy. There was no history of contact with pets or occupational exposure. At presentation, she was hemodynamically stable, febrile, had multiple oral ulcers with left hemiparesis and right upper motor neuron facial palsy. Her hemoglobin was 112 gm/L, total leukocyte count 7.8 × 10^9^/L, platelet count 145 × 10^9^/L and serum creatinine 78 μmol/L. Noncontrast computerized tomography (CT) followed by magnetic resonance imaging of brain revealed a 3 × 3 cm hypodense lesion in right temporo-parietal region with significant peri-lesional edema (Figure 1A & B). An endoscopic examination for dysphagia revealed multiple esophageal ulcers, biopsy from which was positive for cytomegalovirus inclusions. Whole blood quantitative polymerase chain reaction (PCR) for CMV was positive with 17500 copies/mL. Simultaneously, a stereotactic biopsy from the brain lesion was performed. Fungal staining of the material showed septate hyphae, while fungal cultures grew melanin producing filamentous fungi with brown-black colonies. A lactophenol cotton blue mount demonstrated numerous single-celled, broadly clavate to ovoid conidia, 4–9 × 6–10 mm in size, rounded above with truncate bases, borne singly or in small groups on elongated, simple or branched conidiophores or on hyphae. These features confirmed the diagnosis of *Scedosporium apiospermum* (Figure 2). Mycophenolate mofetil and tacrolimus were stopped and she was started on intravenous (i.v.) ganciclovir and i.v. voriconazole. After one week of therapy, she became afebrile with improvement of power in the left upper limb. A repeat CT scan of the head done after one week showed reduction in size of the lesion (Figure 1C). After 3 weeks of therapy, the patient showed significant improvement in the slurred speech and she became ambulatory with support. There was significant reduction in size in repeat CT scan. Repeat CMV PCR done after three weeks was negative and patient was started on valganciclovir prophylaxis and oral voriconazole. The levels of voriconazole were monitored and maintained between 1–5.0 μg/mL for initial 3 months. At the end of two months, she was readmitted with fever and dry cough. Chest radiograph and high resolution CT chest showed thin smooth walled cavitary lesion with surrounding consolidation. A fine-needle aspiration demonstrated acid-fast bacilli, which were confirmed to be *Mycobacterium tuberculosis* on culture. A CT chest was not obtained during the first hospitalization, thus we cannot exclude the possibility of either *Scedosporium* or tuberculosis in the lung at that time. She responded well with rifampicin based four drug antituberculous therapy. During the first month of rifampicin therapy, the median dose of voriconazole needed to be increased to 600 mg per day to maintain its levels in the therapeutic range, while subsequently voriconazole was given at a fixed dose of 200 mg once a day as secondary chemoprophylaxis. At six months of follow-up, she had recovered completely. She was maintaining normal graft function with prednisolone 10 mg once a day with a plan to introduce azathioprine. She continues to take 200 mg of voriconazole, while her antituberculous therapy and valganciclovir have been stopped. She was evaluated for an occult primary immunosuppressive disorder with defective cell mediated immunity, in view of the repeated life threatening infections. There was no hypogammaglobulinemia or CD4 cytopenia and her human immunodeficiency virus (HIV) status was negative.

## Discussion

The risk of infections in transplant recipients is determined by the intensity of epidemiologic exposure to potential pathogens and the net state of immunosuppression. The reasons for higher rates of infection in developing countries include factors like unhygienic conditions, hot and humid environment, overcrowding, high prevalence of endemic infections like tuberculosis, malnutrition, late presentation, unavailability of costly diagnostic investigations like polymerase chain reaction and high cost of life saving antimicrobials [2,3].

CMV is the most frequent pathogen encountered in renal transplant recipients in countries with a temperate climate. CMV infection usually occurs during the second to fourth month period after transplantation; however, delayed onset can be seen in patients on chemoprophylaxis [4,5]. The infection can either be asymptomatic or cause illness of varying severity, depending upon the serological status of the recipient and the donor. In our case, the renal transplant was done at another centre and therefore the pretransplant CMV sero-status of the donor and recipient was not known and she also did not receive CMV chemoprophylaxis. CMV is a potentially preventable disease with the use of prophylactic therapy, especially in high-risk cases, those who receive an induction therapy and who require potent anti-rejection therapy [6] and we feel that the present case would have benefitted by the use of chemoprophylaxis.

Brain abscess is an uncommon infectious complication in renal transplant recipients. Selby et al. [7] described disparate groups of patients with brain abscesses with regard to the timing, susceptibility and predisposition. He found that while fungi (ie, *Aspergillus*, *Candida*, and *Mucorales* sp) contributed to most of the early onset brain abscesses (median 24 days); abscesses that developed late after transplantation (mean 264 days) were caused by non fungal organisms (i.e., *Nocardia* and *Toxoplasma* sp). In another report, *Aspergillus* species were the most commonly isolated organism [8].

*Scedosporium apiospermum* is the anamorph of the ascomycete *Pseudallescheria boydii* which is a genus of the *Ascomycete* order *Microascales. Scedosporium* infections account for about 3% of all fungal infections in post transplant patients with 70% mortality [9]. *Scedosporium* usually causes cutaneous infection; however, sometimes it can cause a disseminated infection [10-12], although isolated brain infection is rare. Being commonly found in heavily polluted environments, agricultural and garden soil, sewer, ditch mud and polluted pond bottoms, drowning is a common cause for brain infection with this fungus [13]. It is commonly misdiagnosed as *Aspergillus* due to its septate hyphal morphology. A search of Pubmed and EMBASE has revealed about eight cases of post renal transplant brain scedosporiosis [11,12,14-19].

Our case did not have any known exposure to above mentioned environmental factors nor had past history of any other fungal infection. She was managed successfully with voriconazole therapy. Voriconazole produces a quick and sustained response with cure rates of up to 57% in immunocompromised subjects with those having brain infection being the worst responders [20]. The infection may persist in the body for long periods of time and has been reported to recur after a second transplant [17,21] and therefore it might be appropriate to give prolonged secondary chemoprophylaxis.

The incidence of post transplant tuberculosis varies from 0.48% in the west [22] to 11.8% in India [23]. Therapeutic response is often satisfactory and drug interactions mandate dose adjustment of other immunosuppression drugs as well as azoles. British thoracic society and European Best Practice Guidelines recommend rifampicin-based four drug therapy for 6 months with blood level monitoring for calcineurin inhibitors [24,25]. Similarly, if the patient is on azoles, the drug levels should be monitored. Although pretransplant evaluation and treatment for latent tuberculosis is recommended, in highly endemic countries like ours, both of these strategies are faced with problems. Due to high endemicity and an increased chance of exposure, evaluation for latent tuberculosis has been shown to be nonspecific and insensitive [26]. Similarly, the treatment of latent tuberculosis by using isoniazid monotheraphy is prone for the development of resistant tuberculosis [27] and there is no consensus on whether latent tuberculosis should be treated in countries otherwise endemic for this infection [28].

## Conclusion

Opportunistic infections in post transplant subjects are very common and require high index of suspicion and proper investigative approach for early diagnosis and treatment. Co-infections can be a diagnostic problem as one infection can easily undermine the existence of the other. In the presence of CMV infection, a co-existent fungal infection needs careful exclusion. Pharmacodynamic monitoring with maintenance of therapeutic levels of immunosuppressive drugs within the prescribed range and tailoring of immunosuppression as per the immunological risk of the patient may provide the long sought balance of adequate immunosuppression with minimal opportunistic infections and malignancies.

Our case appears to be the first in the literature with the triad of CMV, *Scedosporium apiospermum* and *Mycobacterium tuberculosis* infection, all of which were managed successfully. In the present case, since a CT of the chest was not done during the first admission, the probability of pulmonary tuberculosis being there at that time itself cannot be ruled out, though it manifested two months later. This case highlights the importance of getting a microbiological diagnosis from each site involved. One should not assume that if one organism has been isolated, the other manifestations are also because of the same organism. This case also exemplifies the need for tailored immunosuppression for renal transplant recipients to reduce their susceptibility to infections.

### Consent

Written informed consent was obtained from the patient for publication of this case report and any accompanying images. A copy of the written consent is available for review by the Editor of this journal.

## Competing interests

The authors declare no financial or non-financial competing interests.

## Author’s contribution

MR and SG established the diagnosis and managed the patient. MR and RR drafted and edited the manuscript; SM performed the stereotactic biopsy and helped in management of the patient, while HK was instrumental in establishing the microbiological diagnosis. VK and HSK participated in patient management and helped in drafting the manuscript while KLG and VS supervised the management of patient and drafting of manuscript. All the authors have read and approved the manuscript and appointed MR as corresponding author.

## Pre-publication history

The pre-publication history for this paper can be accessed here:

http://www.biomedcentral.com/1471-2334/14/259/prepub
